# Antiseptics in Lying-In Hospitals

**Published:** 1897-02-20

**Authors:** 


### ANTISEPTICS IN LYING-IN HOSPITALS.

An address recently delivered by Dr. Clement Godson before the British Gynæcological Society on "'Antiseptic Midwifery" is of great interest, for it shows on the one hand how great a saving of life results from the careful adoption of modern antiseptic methods, and on the other how the advantages offered by structural and other improvements in hospital buildings may be nullified by want of systematic attention to those details of disinfection without which infectious diseases tend to be carried from case to case within their walls, and thus practically to become endemic.

Speaking of the City of London Lying-in Hospital, he showed what repeated efforts had been made by the committee and the staff to eradicate the septic diseases which years ago used to be the curse of the place, and how fruitless these efforts were until antiseptics were used. When he took office at the hospital he found that women were dying there in the proportion of 1 in 19. In consequence of his representations the place was closed, and elaborate measures were taken to do away with the infection which was presumed to attach to the building. It was cleansed, painted, and coloured throughout; drains were opened and trapped, the old cesspools were filled up with concrete, chlorine fumigations were made use of, and the wards were almost entirely refurnished. The result was for a time good; yet very shortly the mortality rose, and in 1877 the hospital was again closed. Again a large amount of money was spent in cleansing and in structural alterations, and again great improvement in the results was experienced, 287 women being delivered during the remainder of the year with only one death. Yet in the first half of the next year the mortality went up to 1 in 19! Again money was freely spent, but with so small effect that in 1882 the hospital was closed, and it was even urged that the whole place should be pulled down. Instead of this, however, certain alterations and additions were made, and it was reopened. But in five months' time it again had to be closed, in consequence of its unhealthy condition. The combined mortality of the two years 1882-1883 was 1 in 23; and even after the cleansing which then took place the results were not satisfactory, the patients dying at the rate of 1 in every 48 cases during the years 1884-85, and 1 in 44 during 1886, until the hospital was closed again. When, after another cleansing, the hospital was again opened in 1886, the practice of antiseptic midwifery was introduced, the antiseptic used being perchloride of mercury, with this result: that, from the time of reopening, 420 consecutive cases were delivered without a death; that, from that time up to the end of 1896 (ten years and a-half), 4,608 women have been confined within the hospital with only 11 deaths, or 1 in 419; and that, during the last five years of that period the deaths have only been at the rate of 1 in 797.

It is unnecessary to add a single word to this eloquent record, which shows, as no mere argument could show, the efficacy of antiseptic midwifery carried out by the aid of perchloride of mercury; the harmlessness of the process; and, lastly, the inefficacy of other means, even when aided by the most lavish expenditure, in preventing the spread of septic disease in lying-in hospitals.

